# Data as infrastructure: Systematic data curation addressing fundamental data content differences across the UK

**DOI:** 10.23889/ijpds.v9i5.2683

**Published:** 2024-09-18

**Authors:** Chris Orton, Lara Edwards, David Seymour, Monica Jones, Philip Quinlan, Simon Thompson, Carole Goble, Jennifer Quint, Aziz Sheikh

**Affiliations:** 1 Swansea University; 2 Health Data Research UK; 3 University of Leeds; 4 University of Nottingham; 5 University of Manchester; 6 Imperial College London; 7 University of Edinburgh

## Objective and Approach

Health Data Research UK, the UK national institute for health data science, is coordinating efforts alongside national academic partners to streamline data curation at disease, population, and data structure level to enhance data offerings and provide networked data infrastructure supporting whole-UK research.

Due to clinical, coding, and system differences across the constituent countries of the UK, data is often not standardised for whole-UK analyses, creating burden on research teams and leading to long data preparation times in order to run even distributed analyses.

The approach to solve this is multi-faceted, including deploying data curation and cohort creation algorithms into health data providers’ environments, and through the novel integration of federated analytics solutions (such as those piloted through recent national infrastructure programmes) improving data access and research deployment efficiency.

## Results

Standardising data through clinical and structural data curation directly deployed to health data providers creates a framework for whole-UK studies to be readily achievable, and provide the data infrastructure base to integrate new technical federated analytics solutions to deploy and reproduce analytics without unnecessary large scale data migration.

## Conclusions and Implications

Systematic curation of health data within national data providing organisations provides flexibility and choice for researchers in terms of the data they will apply for to answer vital research questions affecting the UK populace. Such advances will improve the quality and efficiency of research for all corners of the UK, and create a community of practice in terms of developing data from health systems to research environments.

